# Functional and structural readouts for early detection of retinal involvement in multiple sclerosis

**DOI:** 10.3389/fnint.2023.1158148

**Published:** 2023-04-17

**Authors:** Khaldoon O. Al-Nosairy, Alexander Duscha, Henrike Buhr, Antonia Lipp, Christiane Desel, Tobias Hegelmaier, Hagen Thieme, Aiden Haghikia, Michael B. Hoffmann

**Affiliations:** ^1^Department of Ophthalmology, University Hospital Magdeburg, Magdeburg, Germany; ^2^Department of Neurology, University Hospital Magdeburg, Magdeburg, Germany; ^3^Center for Behavioral Brain Sciences, Magdeburg, Germany

**Keywords:** MS, optic neuritis (ON), electroretinography (ERG), mfPhNR, mfPERG, OCT

## Abstract

**Introduction:**

The retina, a window into the brain, allows for the investigation of many disease-associated inflammatory and neurodegenerative changes affecting the central nervous system (CNS). Multiple sclerosis (MS), an autoimmune disease targeting the CNS, typically impacts on the visual system including the retina. Hence, we aimed to establish innovative functional retinal measures of MS-related damage, e.g., spatially resolved non-invasive retinal electrophysiology, backed by established morphological retinal imaging markers, i.e., optical coherence tomography (OCT).

**Methods:**

20 healthy controls (HC) and 37 people with MS [17 without history of optic neuritis (NON) and 20 with (HON) history of optic neuritis] were included. In this work, we differentially assessed photoreceptor/bipolar cells (distal retina) and retinal ganglion cell (RGC, proximal retina) function besides structural assessment (OCT). We compared two multifocal electroretinography-based approaches, i.e., the multifocal pattern electroretinogram (mfPERG) and the multifocal electroretinogram to record photopic negative response (mfERG_*PhNR*_). Structural assessment utilized peripapillary retinal nerve fiber layer thickness (pRNFL) and macular scans to calculate outer nuclear thickness (ONL) and macular ganglion cell inner plexiform layer thickness (GCIPL). One eye was randomly selected per subject.

**Results:**

In NON, photoreceptor/bipolar cell layer had dysfunctional responses evidenced by reduced mfERG_*PhNR*_-N1 peak time of the summed response, but preserved structural integrity. Further, both NON and HON demonstrated abnormal RGC responses as evidenced by the photopic negative response of mfERG_*PhNR*_ (mfPhNR) and mfPERG indices (*P* < 0.05). Structurally, only HON had thinned retina at the level of RGCs in the macula (GCIPL, *P* < 0.01) and the peripapillary area (pRNFL, *P* < 0.01). All three modalities showed good performance to differentiate MS-related damage from HC, 71–81% area under curve.

**Conclusion:**

In conclusion, while structural damage was evident mainly for HON, functional measures were the only retinal read-outs of MS-related retinal damage that were independent of optic neuritis, observed for NON. These results indicate retinal MS-related inflammatory processes in the retina prior to optic neuritis. They highlight the importance of retinal electrophysiology in MS diagnostics and its potential as a sensitive biomarker for follow-up in innovative interventions.

## 1. Introduction

Multiple sclerosis (MS) is a chronic inflammatory autoimmune disorder of the central nervous system (CNS). It is the most common neurological disability next to trauma in early to middle adulthood and still incurable (Feigin et al., 2017; Reich et al., 2018). MS is known to influence the visual system by affecting not only the optic nerve, i.e., via optic neuritis (ON), but also the retina. Since the retina is particularly accessible for non-invasive diagnostics, the assessment of retinal structure and function in MS has great potential to advance our understanding of disease-associated inflammatory and degenerative damage. In fact, novel non-invasive functional retinal measures of MS-related damage promise biomarkers of neural damage that are more sensitive than current morphological markers obtained with retinal imaging [ocular coherence tomography (OCT)]. Such highly-sensitive markers are needed for the efficient evaluation of novel therapies, e.g., addressing the immune system (Duscha et al., 2020).

Identifying the site of damage in the retina can serve as a key to detail the underlying pathomechanisms and disease patterns. Damage to the optic nerve, e.g., due to optic neuritis, will affect the output stage, the proximal retina including the so-called inner retina from where the axons of the retinal ganglion cells (RGC) project to form the optic nerve. The input stage, the distal retina, including the so-called outer retina with the photoreceptors, will remain intact. Changes to the responses of the distal retina are therefore likely to be due to pathomechanisms that are independent of damage to the optic nerve. Consequently, if such changes in the distal retina occur in MS, they are likely to be associated with pathomechanisms that are independent of optic neuritis.

The key-tool for the non-invasive and objective assessment of retinal function is the electroretinogram (ERG). Importantly, it can, depending on stimulation mode and response component, help to differentiate whether damage affects proximal or distal retinal damage (Heckenlively and Arden, 2006). For the ERG to flash stimulation (flash ERG), early components of the flash ERG are more likely to arise from the distal retina, such as the a-wave that is dominated by photoreceptor activity. Later components of the flash-ERG are more likely to arise from the proximal retina, such as the photopic negative response (PhNR) that is dominated by RGC activity. For the ERG to pattern stimulation (PERG), a positivity around 50 ms (P50) is dominated by responses from the RGC bodies, a negativity around 95 ms (N95) is likely to arise from the axons (Al-Nosairy et al., 2021a). Both flash- and pattern-ERG can be combined with the multifocal stimulation technique. The multifocal approach allows for quasi-simultaneous stimulation at distinct visual field locations in order to determine the visual field topography of retinal function (Sutter and Tran, 1992; Sutter, 2001) within a short time window. It should be noted that, in terms of generators, the multifocal flash ERG (mfERG) is more bipolar-cell dominated than the conventional flash ERG, such that the bipolar cells also have a contribution to early components (Hood et al., 2002). This is reflected by a different peak terminology for the mfERG, i.e., N1 and P1 - instead of the conventional a-wave and b-wave terminology (Hoffmann et al., 2021). Similarly, mfPERG equivalents of the PERG P50 and N95 are termed differently, i.e., P1 and N2, although P50 and P1 as well as N95 and N2 seem to have similar generators (Bach et al., 2018). In short, the mfERG and the mfPERG are two independent tools to uncover RGC malfunction, via the PhNR and the P1 or N2, respectively.

In a previous study, we pioneered mfPERG readouts in MS patients with and without a history of optic neuritis (HON and NON, respectively). We reported foveal RGC axonal dysfunction (N2 amplitude reduction), independent of the history of optic neuritis (Al-Nosairy et al., 2021b) and suggested a hyper-excitability associated with inflammatory processes in HON (P1 peak time reduction). The mfERG variation to record the PhNR, i.e., mfERG_*PhNR*_ (Al-Nosairy et al., 2020b), a faster and more robust electrophysiological technique than the mfPERG, has not been applied to MS yet. Still, its potential to indicate RGC dysfunction has been demonstrated previously in patients with RGC-loss induced by the frequent eye disease glaucoma (Viswanathan et al., 1999, 2001; Al-Nosairy et al., 2020a,2021b). Hence, a comparative assessment of MS-related retinal damage with mfPERG, mfERG_*PhNR*_, and OCT appears warranted in order to uncover effective retinal biomarkers of MS. Such multimodal assessments are intended to uncover the pattern of MS-related damage and to improve disease diagnostic and follow-up.

With a combined multimodal structural (OCT) and functional assessment (mfERG_*PhNR*_ and mfPERG) of retinal integrity, we here aim to improve the retinal readouts for a differential assessment of proximal and distal retinal structural and functional integrity in two MS cohorts with a different history of optic neuritis, i.e., HON and NON. Specifically, we addressed two key questions: (i) What is the most sensitive biomarker of retinal damage in MS patients and does it depend on the history of optic neuritis? (ii) Is the distal retina involved in neurodegenerative process of MS in the absence of optic neuritis?

## 2. Materials and methods

This study followed the tenets of the declaration of Helsinki and the protocol approved by the ethical committee of the Otto-von-Guericke University of Magdeburg, Germany (No 73/21, 2021). After obtaining informed consent from each participant, this prospective observational study was conducted at the Departments of Ophthalmology and Neurology, University Hospital Magdeburg, Germany.

### 2.1. Participants

All participants underwent rigorous neurological and ophthalmological examinations including expanded disability status scale [EDSS, for clinical disability quantification in MS (Kurtzke, 1983)], visual acuity with refraction correction, and visual field testing (VF). Exclusion criteria were any other systemic, ophthalmic diseases that might influence electrophysiological or neurological measurements. One eye was selected per subject, if both eyes met the eligibility criteria.

HC. 20 healthy subjects with normal ophthalmic and neurological examinations participated in this study.

MS. 37 patients with a confirmed diagnosis of clinically definite relapsing-remitting MS according to the revised McDonald criteria (Thompson et al., 2018) were enrolled in this study. HON patients (*n* = 20): Patients with a single history of optic neuritis at least 1 year ago. NON patients (*n* = 17): Patient without evidence of a clinical picture of optic neuritis [normal visual evoked potential (VEP) peak time except for three cases with prolonged peak time >120 ms]. Because of the extensive nature of this study, a few patients were unable to complete all planned measurements and dropped from ERG recording or OCT measurements as detailed below in the table of functional and structural measures.

### 2.2. Functional readouts: electrophysiological recordings

#### 2.2.1. Conventional electrophysiology

Macular and optic nerve functions were assessed using standard electrophysiology measures including pattern reversal visual evoked potentials (prVEP) and pattern electroretinogram (PERG). See Table 1 for the specific parameters used.

**TABLE 1:** VEP and PERG recordings.

	prVEP^1^	PERG^2^
Recording device	EP2000 Evoked potentials system (Bach, 2005)	
Size of stimulus	0.25° and 1°	1° and 15°
Stimulus type	Checkerboard	Checkerboard
Stimulation	Pattern reversal (2 reversals per second)	-ssPERG^3^: 15 rps (equivalent to 7.5 Hz) for -tPERG^4^: 4 rps (equivalent to 2 Hz)
Chromacity	Achromatic	Achromatic
Contrast; Mean luminance	98%; 50 cd/m^2^	98%; 50 cd/m^2^
Recording settings	50 k amplified, band-pass filtered (0.3–100 Hz) and at 1 kHz digitized. Signals exceeding ± 90 μV were online rejected. digital 40 Hz cutoff low pass	50 k amplified Band-pass filtered (1–100 Hz) and at 1 kHz digitized Signals exceeding ± 90 μV were online rejected. digital 40 Hz cutoff low pass
Repetitions	Twice (A-B-B-A scheme)	Twice (A-B-B-A scheme)
Electrodes and eye	Gold cup electrodes at Oz, with reference to Fz and ground electrode at Fpz. Monocular recording	Gold cup electrodes placed 5 mm below the lower eyelid, referenced to the ipsilateral outer canthus and a ground electrode placed at Fpz Binocular recording
Viewing distance	36 cm	36 cm
Reporting	P100 amplitude and peak time	ssPERG -1 and 15° amplitudes -ssPERG ratio: small/big checkerboard size amplitude tPERG: -P50 and N95 -Ratio of P50 and N95: small/big checkerboard size amplitudes

Following International Society for clinical Electrophysiology of Vision (ISCEV)^1^pattern reversal Visual evoked potential [prVEP (Odom et al., 2016)] and ^2^Pattern electroretinogram [PERG standards (Bach et al., 2013)]. ^3^Steady state Pattern electroretinogram (ssPERG) (Al-Nosairy et al., 2020c). ^4^Transient pattern electroretinogram (tPERG).

#### 2.2.2. Multifocal stimulations

To topographically assess retinal function using ERG measures, we adopted multifocal stimulation, a technique developed by Sutter and Tran (1992) and Sutter (2001), utilizing binary m-sequence stimulation. This technique allows quasi-simultaneous stimulation of the visual field and enables extraction of individual locations from the summed response within a short recording time. This is facilitated by the mathematical independence of stimulation sequences, i.e., binary m-sequence, where choosing different starting points in the m-sequence allow uncorrelated of responses from each other and hence triggering a localized response within the retina, see (Hoffmann, 2008) for further details. This enabled obtaining the multifocal ERG of a flash stimulus (mfERG), and multifocal PERG to a pattern stimulus, i.e., multifocal pattern electroretinogram (mfPERG), see Table 2. It should be noted that in the present publication we use the term “photoreceptors/bipolars” to refer to both generators of the N1 of the mfERG_*PhNR*_, i.e., the cone photoreceptors and the bipolar cells (Heynen and van Norren, 1985; Bush and Sieving, 1994).

**TABLE 2:** Multifocal retinal function recordings.

	mfERG_PhNR_^1^	mfPERG^2^
Stimulus delivery and recording	VERIS Science^3^	VERIS Science
Size of stimulated field	46°	45°
Individual VF locations	5	36
Eccentricities	Central ring: 4.8° Outer ring (4 fields): 23.1°	ring1: 0.0–3.6, ring2: 3.6–7.6 ring3: 7.6–14.3; ring4: 14.3–22.7°
Stimulus type	Bright light	4 × 4 checkerboard
	Flash	Pattern reversal (pr)^4^
	(0) state: no flash	(1) state: Pattern 1
	(1) state: flash	(2) state: Pattern 2
	9 interleaving frames	2 frames (Hoffmann and Flechner, 2008)
Traces	Figure 1A	Figure 1B
Chromacity	Achromatic	Achromatic
Mean luminance	104 cd/m^2^	56 cd/m^2^
m-sequence; step duration	2^9^-1; 13.3 ms	2^14^-1; 26.6 ms (Hoffmann and Flechner, 2008)
Recording settings	The signals amplified by 100 K^5^ band pass filtered 3–300 Hz and digitized at 1200 Hz. Traces were then digitally filtered (3–45 Hz)^6^	
Response analysis	1st order kernel	1st slice of the 2nd order kernel
Repetitions	6 times	3 times
Components, origin, nomenclature	-N1,1st negativity: cone “photoreceptors/bipolars”^7^ (Heynen and van Norren, 1985; Bush and Sieving, 1994). -P1, 1st positivity: cone bipolar cells, horizontal cells (Sieving et al., 1994) -mfPhNR, 2nd negativity: retinal ganglion cells (Viswanathan et al., 1999).	-P1^8^, 1st positivity: retinal ganglion cell bodies (Bach et al., 2018) -N2^8^, 2nd negativity: optic nerve head (Bach et al., 2018)
Reporting	Individual waves and mfPhNR ratio^9^: mfPhNR/P1 wave, both summed across 2 eccentricities and whole stimulated field	P1 and N2 summed across 4 eccentricities and whole stimulated field

^1^Further details: (Al-Nosairy et al., 2020a,2021b). ^2^Further details: (Al-Nosairy et al., 2021b). ^3^VERIS 5.1.12XScience (EDI: Electro-Diagnostic Imaging, Redwood City, CA, USA). ^4^Contrast-inverted version of pattern 1; pattern reversal is extracted by extracting the 2nd order kernel. ^5^Grass Model 12, (Astro-Med, Inc., West Warwick, RI, USA). ^6^Analysis with Igor (IGOR Pro, WaveMetrics, Portland). ^7^“photoreceptors/bipolars” will be used to describe the two generators of the mfERG_PhNR_-N1 component, i.e., the cone photoreceptors and the bipolar cells (Heynen and van Norren, 1985; Bush and Sieving, 1994). ^8^P1 and N2 are quite similar to P50 and N95, respectively, of transient PERG responses but due to temporal differences, we stick with this nomenclature. ^9^mfPhNR ratio is calculated based on findings of previous study (Al-Nosairy et al., 2020b) and ISCEV recommendation (Frishman et al., 2018), i.e., to determine whether mfPhNR reduction is due to proximal or distal generators. mfERG_PhNR_, multifocal electroretinogram to record photopic negative response; mfPhNR, photopic negative response of mfERG_PhNR_; mfPERG, multifocal pattern electroretinogram; VF, visual field.

The stimuli for all ERG recordings were presented at a frame rate of 75 Hz on a monochrome monitor (MDG403; Philips, Amsterdam, Netherlands; P45 phosphor). Skin gold-cup electrodes were filled with conductive paste (Ten20, WEAVER and Company, Aurora, CO, USA) and attached after skin cleaning with a paste (skinPure, NIHON KODEN Corporation, Tokyo, Japan) to reduce the resistance of the skin below 5 kOhm. Only mfERG_*PhNR*_ recordings were conducted after Pupil dilatation (to at least 7 mm). For mfPERG and mfERG_*PhNR*_, traces from right eyes were left-right flipped to match stimulated visual fields of traces recorded from left eyes of other participants. Refractive correction was applied for 36 cm viewing distance.

**FIGURE 1:**
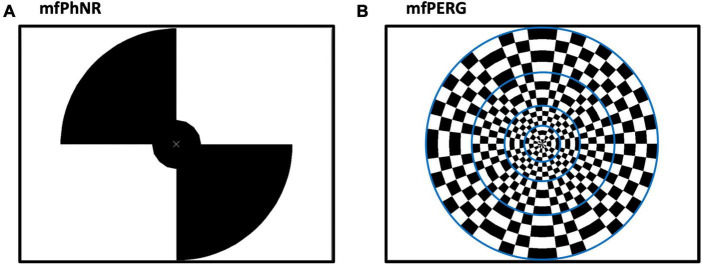
**(A)** Multifocal electroretinogram to record photopic negative response (mfERG_PhNR_) stimulus spanning two eccentricities with five stimulated fields (snap shot with two peripheral locations “on”, and two plus the central “off”) and **(B)** Multifocal pattern electroretinogram (mfPERG) spanning four eccentricities with a total of 36 stimulated fields. Blue lines are not seen by the patient but to graphically delineate different eccentricities.

### 2.3. Structural assessment: optical coherence tomography (OCT)

Retinal structure was assessed using a spectral domain OCT device (Heidelberg Spectralis^®^, Heidelberg Engineering, Heidelberg, Germany) for the macula and the optic disc.

Macula. The macula scan of 61 vertical B scans (each with 768 A-Scans, automatic real-time = 13 frames) covered an angle of 30° × 25°. Early Treatment Diabetic Retinopathy Study (ETDRS) protocol was used to depict the thickness analysis for retinal layers. Two measures of interest were mainly analyzed at the proximal and distal layers of the retina. The proximal layer assessing RGC was determined by ganglion cell layer and inner plexiform layer (GCIPL) while the distal layer assessing photoreceptor layer was determined by outer nuclear layer thickness (ONL). Both measures were averaged within the central, parafoveal (3 mm) and perifoveal (9 mm) rings of the ETDRS layout.

Optic disc. The proximal layer including the RGC axons was also assessed by measurements of the peripapillary retinal nerve fiber layer thickness (pRNFL) from a 3.5 mm circle scan centered on the optic disc (12° diameter). The pRNFL was calculated from the averaged (pRNFL G), papillomacular bundle (pRNFL PMB), and temporal or nasal (pRNFL T or N) sectors thickness.

### 2.4. Analysis

Statistical tests were conducted using “R”(R Core Team, 2013) after checking normality. ANOVA or Kruskal Wallis were then applied accordingly with *post hoc* comparisons. Area under curve of Receiver operating characteristics (AUC of ROC) determined the tests with higher performance in detection of ON status and AUC of different tests were also compared to establish a significant difference. Adjustment of *P*-values for multiple testing was performed with the Sidak correction.

## 3. Results

### 3.1. Functional readouts of retinal damage in MS

**FIGURE 2:**
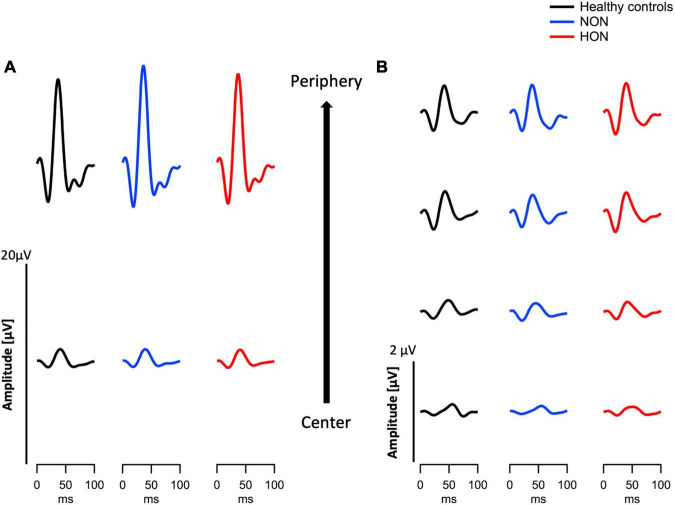
Eccentricity-based depiction of grand means traces of two modes of electroretinography in the three different groups, healthy, MS with/without optic neuritis, HON vs. NON, respectively. **(A)** Multifocal electroretinogram (mfERG_PhNR_) traces summed in two eccentricities. **(B)** Multifocal pattern electroretinogram (mfPERG) traces spanning four eccentricities.

For a qualitative overview the grand mean traces of mfERG_PhNR_ and mfPERG are shown in Figures 2A, B for each participant group. In a subsequent quantitative analysis of the peak times and amplitudes based on the individuals’ traces, we addressed the impact of MS on the retina at both distal and proximal retina.

**FIGURE 3:**
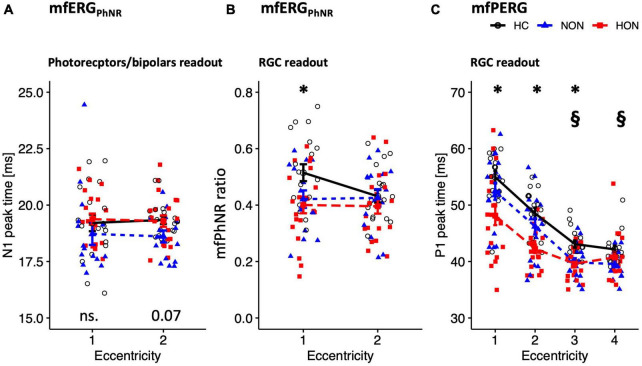
Functional readouts eccentricity-based analysis depicted as mean ± SEM. **(A,B)** mfERG_PhNR_ stimulation spanning two eccentricities: **(A)** N1 peak time. **(B)** mfPhNR ratio. **(C)** mfPERG P1 peak times across four eccentricities. For legends, see Table 3. *P*-values of comparative analysis across the three groups are displayed along the *x* axis for each measure. *Indicates significant HON vs. HC differences. §Indicates significant NON vs. HC differences.

The distal retinal readouts generated by cone photoreceptors/bipolars, show abnormalities in the ERG responses of MS patients without history of optic neuritis (NON) in comparison to healthy controls (HC). This might indicate early retinal involvement independent of optic nerve status in MS. Specifically, the peak time of the mfERG_*PhNR*_-N1 (summed for both rings), was reduced by 0.90 ± 0.31 ms in NON vs. HC, *p* = 0.021, see Figure 3A for individual rings’ peak times. The mfERG_*PhNR*_-N1 amplitudes were, nevertheless, not significantly different between groups.

Since NON demonstrated “photoreceptors/bipolars” (as defined in section “Materials and methods) involvement in MS, it was intriguing to ascertain the proximal retinal responses at the level of the retinal ganglion cells (RGC). Indeed, RGC responses were affected in NON as evidenced by both the mfERG_PhNR_ and mfPERG (Figures 3B, C). The mfPhNR peak time was reduced by less than 3 ms in the central retina (5°) in NON vs. healthy, *p* = 0.025. The mfPERG-P1 peak times were also reduced by ≥2.5 ms in the outer 2 rings of the retina in NON vs. HC, *p* < 0.05. At this retinal level, mfERG_PhNR_ (P1 and mfPhNR) and mfPERG (P1 and N2) amplitudes were normal in NON.

Further, MS patients with optic neuritis (HON) demonstrated abnormal functional parameters at the level of RGC as evidenced by both the mfERG_PhNR_ and mfPERG (Figures 3B, C). The mfPhNR ratio was reduced by 0.11 ± 0.04 in the central 5° retina of HON vs. HC (Figure 3B), *p* = 0.022, and the mfPERG-P1 peak times were reduced by 3.5–6.8 ms in the inner 3 rings (0–17°) of retina in HON vs. HC, (Figure 3C), *p* < 0.01.

Although the topographical analysis of ERG responses allows for the stratification and delineation of the extent of the retinal damage in the target patient groups, we also assessed global retinal responses by using ssPERG and tPERG. Here, only the ssPERG-ratio and the tPERG-ratio of the P50 component (as defined in section “Materials and methods) were statistically different as evident from an ANOVA across groups, *F*(2,50) = 7.9, *P* = 0.002 and *F*(2,49) = 3.4, *P* = 0.042, respectively. *Post hoc* tests specified that the difference was driven by a reduction for NON vs. HC [*P* < 0.05 (Table 3)].

**TABLE 3:** Functional and structural measures.

		HC	NON	HON	ANOVA	P	*Post hoc* testing^†^		
			Mean ± SD				HC vs. NON	HC vs. HON	NON vs. HON
**mfERG_PhNR_**		19	20	15					
Peak time [ms]	*N1 summed*	19.43 ± 0.8	18.56 ± 1.1	19.29 ± 0.9	f(2,51) = 4.4	**0.017**	**0.021**	0.85	0.06
	*P1 ring 1* ^‡^	40[2.5]	38.3 [3.3]	39.6[1.7]	H(2) = 6.1	**0.047**	0.053	0.62	0.16
	mfPhNR ring 1^‡^	64.2[23.3]	61.7[12.5]	63.3[20]	H(2) = 7.6	**0.022**	**0.025**	0.58	0.062
mfPhNR Ratio	Ring 1	0.51 ± 0.1	0.42 ± 0.1	0.4 ± 0.1	f(2,51) = 4.3	**0.019**	0.11	**0.022**	0.95
**mfPERG**		17	20	17					
P1 peak time [ms]	Ring 1	54.95 ± 4.6	52.45 ± 6.3	48.12 ± 7.5	f(2,51) = 5.6	**0.006**	0.58	**0.006**	0.12
	Ring 2	48.53 ± 4.2	46.23 ± 6.0	42.25 ± 3.1	f(2,51) = 9	**<0.001**	0.15	**<0.001**	**0.03**
	Ring 3	43.09 ± 2.7	39.85 ± 3.1	39.63 ± 2.7	f(2,51) = 8.3	**<0.001**	**0.005**	**0.001**	0.99
	Ring 4^‡^	40.8[11.7]	39.2[8.3]	40.0[18.1]	H(2) = 7.2	**0.028**	**0.031**	0.09	0.5
**ss/tPERG**		17	20	16					
ssPERG	Ratio	1.04 ± 0.2	0.76 ± 0.2	0.89 ± 0.2	f(2,50) = 7.1	**0.002**	**0.001**	0.113	0.207
tPERG	P50 ratio	0.95 ± 0.2	0.79 ± 0.2	0.85 ± 0.2	f(2,49) = 3.4	**0.042**	**0.039**	0.295	0.66
**Macula**		20	19	14					
GCIPL [μm]	Center	44.5 ± 15.1	36.2 ± 11.0	32.7 ± 4.9	f(2,50) = 5.6	**0.006**	0.115	**0.006**	0.759
	Parafoveal	94.2 ± 9.3	83.3 ± 16.5	73.3 ± 15	f(2,50) = 11.5	**<0.001**	0.073	**<0.001**	0.125
	Perifoveal	62.2 ± 5.13	60.7 ± 8.03	54.5 ± 8.0	f(2,50) = 6.4	**0.003**	0.96	**0.004**	**0.045**
**Optic disc**		20	19	15					
pRNFL [μm]	Averaged	97.7 ± 7.6	91.1 ± 10.8	83.8 ± 13.6	f(2,51) = 7.9	**0.001**	0.234	**<0.001**	0.16
	PMB	52.7 ± 9.0	46.4 ± 10.4	39.6 ± 10.6	f(2,50) = 8.3	**<0.001**	0.2	**<0.001**	0.15
	Temporal	68.7 ± 11.9	62.2 ± 15.2	52.3 ± 13.9	f(2,51) = 7.2	**0.002**	0.43	**0.001**	0.11

Italics variables denoted response from distal retinal (photoreceptors/bipolars indices) in contrast to non-italics for proximal retina (retinal ganglion cell indices). ^‡^Non-parametric tests and data reported in median (range). ^†^P-values are corrected for multiple testing using Sidak method. Bold values significant the p-values. N/HON, no/history of optic neuritis; multifocal/electroretinogram to record photopic negative response (mfERGPhNR) and the photopic negative response component (mfPhNR); mf/PERG, multifocal/pattern electroretinogram; ss/t PERG, steady state/transient PERG; GCIPL, ganglion cell inner plexiform layer thickness; pRNFL, peripapillary retinal nerve fiber layer thickness; PMB, papillomacular bundle of pRNFL thickness.

### 3.2. Structural readouts of retinal damage in MS

After elucidating functional retinal involvement, we established the structural involvement in MS, particularly NON. This might serve to detect MS-related retinal changes early and to establish novel biomarkers for follow-up.

Although we showed, as detailed above, functionally abnormal retina at the photoreceptors/bipolars level, the thickness of the distal retinal was structurally intact in both MS groups as evidenced by comparable outer retinal nuclear thicknesses (ONL) between MS and HC.

**FIGURE 4:**
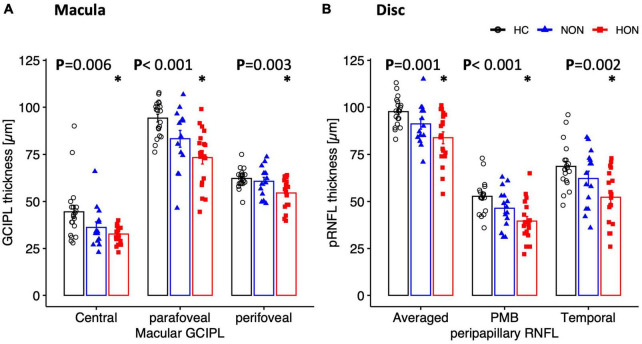
Structural measures of macula **(A)** and peripapillary **(B)** areas depicted as mean ± SEM. Ganglion cell inner plexiform layer thickness of the macula is significantly reduced in HON in the three scanned regions. Peripapillary retinal nerve fiber layer thickness (pRNFL), mainly the temporal sector and papillomacular bundle as well as the averaged thickness, is significantly reduced only in HON. *Indicates significant HON vs. HC differences.

At the proximal retina, RGC thickness was reduced only in HON both at the macula and optic disc. Here, macular GCIPL was reduced by 7.1 ± 3.6 μm, e.g., centrally, in HON vs. HC, *p* < 0.006 (see Table 3 and Figures 4A, B). Averaged peripapillary RNFL was also 13.9 ± 3.5 μm lower in HON in comparison to HC, *p* < 0.001.

### 3.3. Classification based on AUC

Based on AUC of ROC analysis, both functional and structural measures had good performance in detecting MS damage in comparison to healthy eyes. Here, the AUC ranged from 70 to 80% for both functional and structural measures and an absence of significant difference between modalities, i.e., structural vs. electrophysiological readouts (Table 4). Since functional readouts provided superior performance for detection of NON status, it is of interest whether ERG measures might provide higher sensitivities to classify HC vs. NON. ROC analysis did not show higher AUC to detect NON and AUCs were comparable to the detection of MS in general, but it needs to be kept in mind that the sample size fell short of that for the merged NON and HON data-set.

**TABLE 4:** Diagnostic performance of functional and structural measures.

Parameters		AUC	Accuracy	Sensitivity	Specificity
**mfERG_PhNR_**	mfPhNR ratio ring1	0.71	0.63	0.51	0.84
**mfPERG**	Ring1	0.71	0.63	0.49	0.94
P1 peak time	Ring 2	0.76	0.74	0.78	0.65
	Ring 3	0.80	0.67	0.51	1.0
	Ring 4	0.72	0.65	0.51	0.94
**Macula**	Center	0.77	0.79	0.91	0.60
GCIPL	Parafovea	0.81	0.72	0.61	0.90
	Perifovea	0.70	0.70	0.58	0.85
pRNFL	Average	0.76	0.67	0.50	0.95
	PMB	0.77	0.75	0.76	0.74
	Temporal	0.71	0.67	0.56	0.85

For abbreviations, see Table 3. AUC, area under curve.

## 4. Discussion

In this study, we used two main ERG techniques employing multifocal stimulation, mfERG_PhNR_ and mfPERG, to scrutinize functional retinal parameters for distinct visual field locations in MS alongside structural measures of macula and optic disc. Being integrated for the first time in MS research, mfERG_PhNR_ showed abnormal photoreceptors/bipolars responses (distal retina) only in NON but dysfunctional RGC in both HON and NON. In accordance, the mfPERG recordings demonstrated abnormal RGC responses (proximal retina) in both groups. Structural indices of the OCT were only affected in HON at the level of the proximal retina.

### 4.1. Distal retinal damage in MS at the photoreceptors/bipolars layer level

The current literature has not yet resolved whether MS damage is evident in distal retinal layers and whether there is a potential primary involvement at this level of the retina. We examined functional and structural retinal measures and report, in contrast to a previous study (Hokazono et al., 2013), abnormal photoreceptors/bipolars function, but preserved structural integrity in NON. Saidha et al. (2011), in line with our finding, demonstrated abnormal distal retinal responses, i.e., reduced mfERG-P1 peak time in NON, but found, in contrast to our and others’ findings (Hanson et al., 2018; Al-Nosairy et al., 2021b), a thinned distal retina. In contrast to the present study, others have also demonstrated abnormal distal retinal layer responses in HON, i.e., reduced mfERG-N1 and -P1 peak times (Filgueiras et al., 2019) or delayed mfERG-P1 peaks (Hanson et al., 2018). Whether the reduced peak times of the distal retina are due to hyperexcitable inflammatory neurons or due to loss of specific cell components generating the responses (Ikeda et al., 1989; Hood et al., 2002; Filgueiras et al., 2019) is still unresolved. While our studies clearly support the early involvement of the distal retina, further research is required to obtain clarity about the sequence of events. Specifically, prospective longitudinal studies might have the potential to achieve this. This would contribute to our understanding of eye involvement in MS and facilitate identification of novel biomarkers for early identification and management of MS.

### 4.2. Proximal retinal damage at the RGC level

In contrast to the above disagreement on the influence of MS on the function of the distal retina, there is consensus about the involvement of the proximal retina in MS. In our previous investigation (Al-Nosairy et al., 2021b), employing mfPERG and OCT, abnormal RGC axons were demonstrated both functionally (reduced mfPERG-N2 amplitude) and structurally (reduced pRNFL-PMB thickness) in NON, and these changes were also evident in HON besides reduced macular ganglion cell layer (GCIPL). In the present study, NON showed only RGC functional changes whereas both RGC functional and structural changes were evident in HON. The variance of these results might be associated with different extents of MS-induced retinal damage and retinal inflammation in patient cohorts of the two studies. Yet, the different studies convey a coherent message: proximal retinal and optic disc involvement in both HON, reflected by structural (Hokazono et al., 2013; Al-Nosairy et al., 2021b) and functional measures (Holder, 1991; Wang et al., 2012; Hokazono et al., 2013; Al-Nosairy et al., 2021b) and in NON, also reflected by structural (Hokazono et al., 2013; Sriram et al., 2014; Al-Nosairy et al., 2021b) and functional measures (Klistorner et al., 2009; Wang et al., 2012; Al-Nosairy et al., 2021b).

### 4.3. Clinical relevance and future directions

The extensive nature of functional and structural investigations of retinal function in MS in our and related studies are driven by the search for the best measure in terms of diagnostic performance and hence biomarkers. The selection of this measure would allow for a shortening of the examination procedures in clinical practice.

The multimodal approach employed in this study allows for a detailed understanding of the damage patterns inflicted by MS on the eye. This was evident by demonstrating a topographical and hierarchical variance that depended on optic neuritis status. This could be implemented in clinical practice in order to understand and manage MS-related vision complaints. As shown in our present study, there was comparable diagnostic performance of both functional and structural indices in MS with AUCs ranging between 70 and 80%, which is in accordance with previous reports (Chua et al., 2022; Piedrabuena and Bittar, 2022). Based on these findings and our results, ERG, and OCT appeared to be complementary and each appeared to provide a different and integral dimension of MS damage. However, functional measures were the only affected retinal readouts in MS that were not related to optic neuritis. This might open the possibility to utilize ERG measures, particularly peak times shortening, for early MS diagnosis and therapy monitoring. While our studies clearly suggest the early involvement of the distal retina, further research is required to obtain clarity about the sequence of events. Specifically, prospective longitudinal studies might have the potential to achieve this. This would contribute to our understanding of eye involvement in MS and further facilitate identification of novel biomarkers for early identification and management. Further, it is of interest to broaden the research to obtain an understanding of the link between inflammation-related changes in MS and retinal effects by studying the interrelation of retinal indices (functional and structural) and brain lesions in MS as assessed by imaging. For example, we recently employed OCT and diffusion MRI as surrogate biomarkers for structural retinal and brain damage in MS and demonstrated that an episode of optic neuritis (HON) induces long-term structural damage at both levels, the retina and the visual brain, specifically the optic radiation. This revealed MS-related retrograde and anterograde neuroaxonal degeneration in inflammatory autoimmune responses involving the visual pathway (Pawlitzki et al., 2020).

### 4.4. Conclusion

Electrophysiological measures of vision further our understanding of mechanisms and pathogenesis of retinal and vision related disorders, specifically MS. In fact, the present study demonstrated dysfunction in early MS that affects the distal retina. It indicates that MS might cause primary distal retinal pathologies, i.e., at the level of the photoreceptor-bipolar cell complex, which are independent of optic nerve damage. These findings mandate further research in terms of appropriate longitudinal studies.

## Data availability statement

The raw data supporting the conclusions of this article will be made available by the authors, without undue reservation.

## Ethics statement

The studies involving human participants were reviewed and approved by the ethical committee of the Otto-von-Guericke University, Magdeburg, Germany (No 73/21, 2021). The patients/participants provided their written informed consent to participate in this study.

## Author contributions

KA-N, AD, MH, and AH: study concept and design. HB and AL: data collection. KA-N and HB: data analysis and interpretation of results. KA-N: manuscript draft. KA-N, AD, HB, AL, CD, TH, HT, AH, and MH: critical revision, comments, and manuscript revision. All authors contributed to the article and approved the submitted version.
